# Supplementary material for: Enhanced Angiogenesis in HUVECs Preconditioned with Media from Adipocytes Differentiated from Lipedema Adipose Stem Cells In Vitro
Source: Int J Mol Sci. 2023 Sep 1;24(17):13572. doi: 10.3390/ijms241713572 (PMC10487727; doi:10.3390/ijms241713572)
Supplement: Supplementary file 1 [file ijms-24-13572-s001.zip › ijms-2564723-supplementary.pdf]

Supplementary Material

Enhanced Angiogenesis in HUVECs Preconditioned with Media from Adipocytes Differentiated from Lipedema Adipose Stem Cells In Vitro

Sara Al-Ghadban [1,*], Samantha G. Walczak[1], Spencer U. Isern[1], Elizabeth C. Martin[2], Karen L. Herbst[3] and Bruce A. Bunnell[1,*]

1 Department of Microbiology, Immunology and Genetics, University of North Texas Health Science Center, Fort Worth, TX, USA

2 Department of Medicine, Section of Hematology and Oncology, Tulane University

3 Total Lipedema Care, Tucson, AZ, USA; kaherbst@gmail.com

* Correspondence: sara.al-ghadban@unthsc.edu (S.A.-G.); bruce.bunnell@unthsc.edu (B.A.B.)

Supplementary Figures

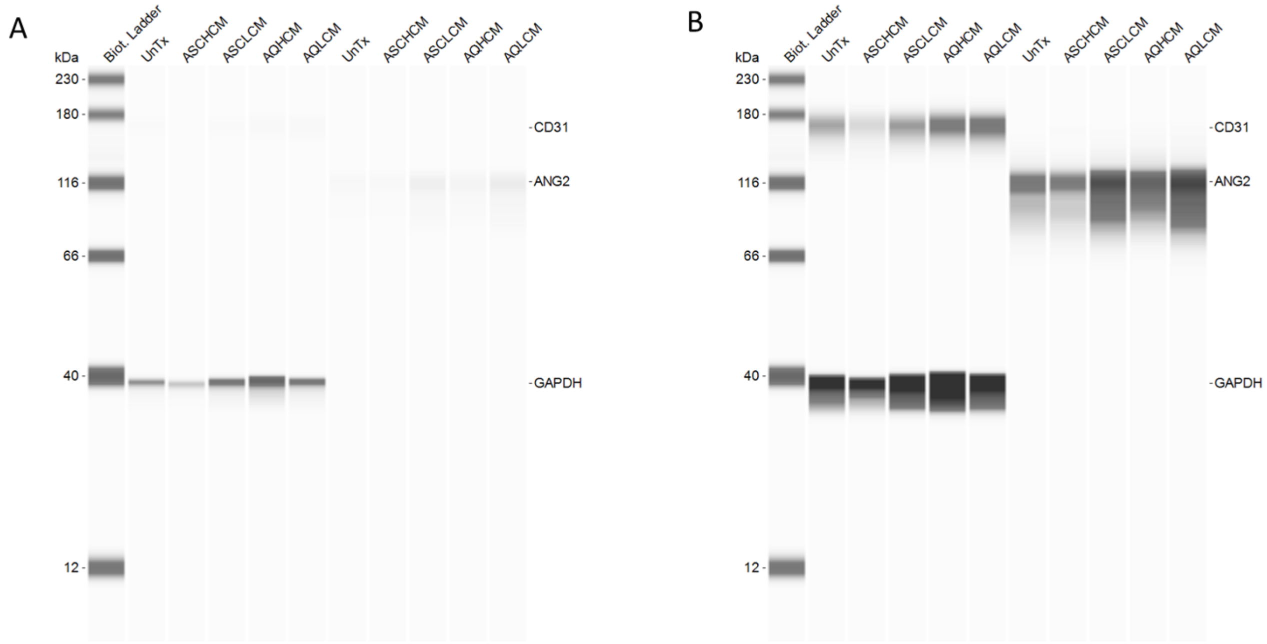

Figure S1. Western Blot showing the expression of CD31, Angiopoietin (ANG2) and GAPDH in HUVECs treated with conditioned media in 2D monolayer culture. The samples were run on a 12-230 kDa capillary kit. A-B are different exposures of the same run.

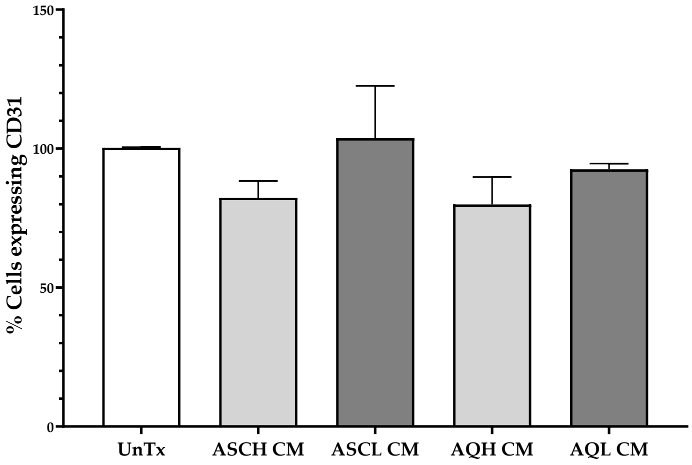

Figure S2. Flow cytometry analysis showing similar expression levels of CD31 in controls and HUVECs treated cells (n=3).

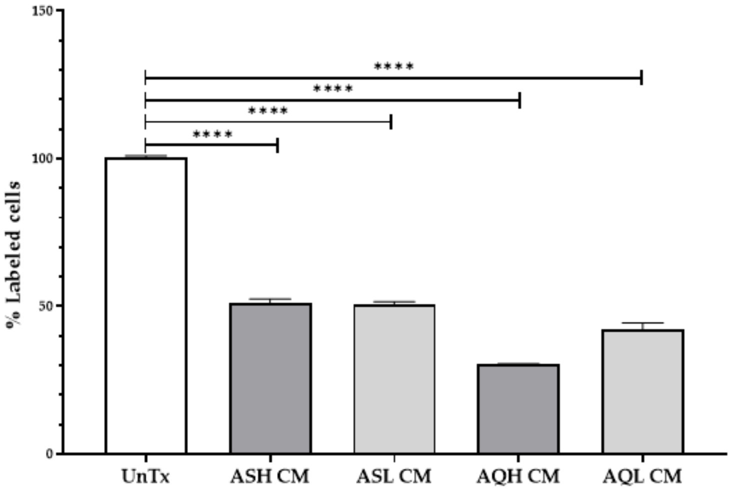

Figure S3. Histogram representing the percentage of calcein transfer into unlabeled HUVECs after co-culture (single cells, n=3). The values are the mean SEM. **** $p < 0.0001$.

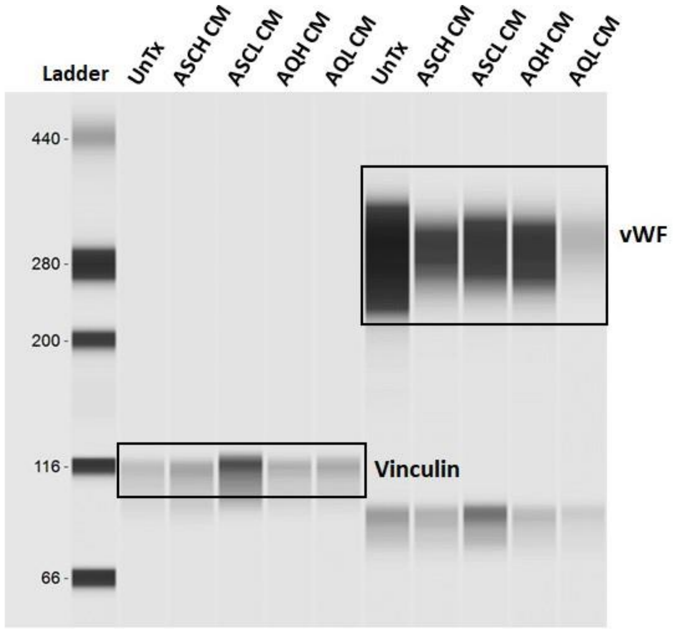

Figure S4. Western Blot analysis showing vWF (300 kDa) and Vinculin (116 kDa) protein expression in an assembled gel-like image view. Expression levels vWF protein were normalized Vinculin.

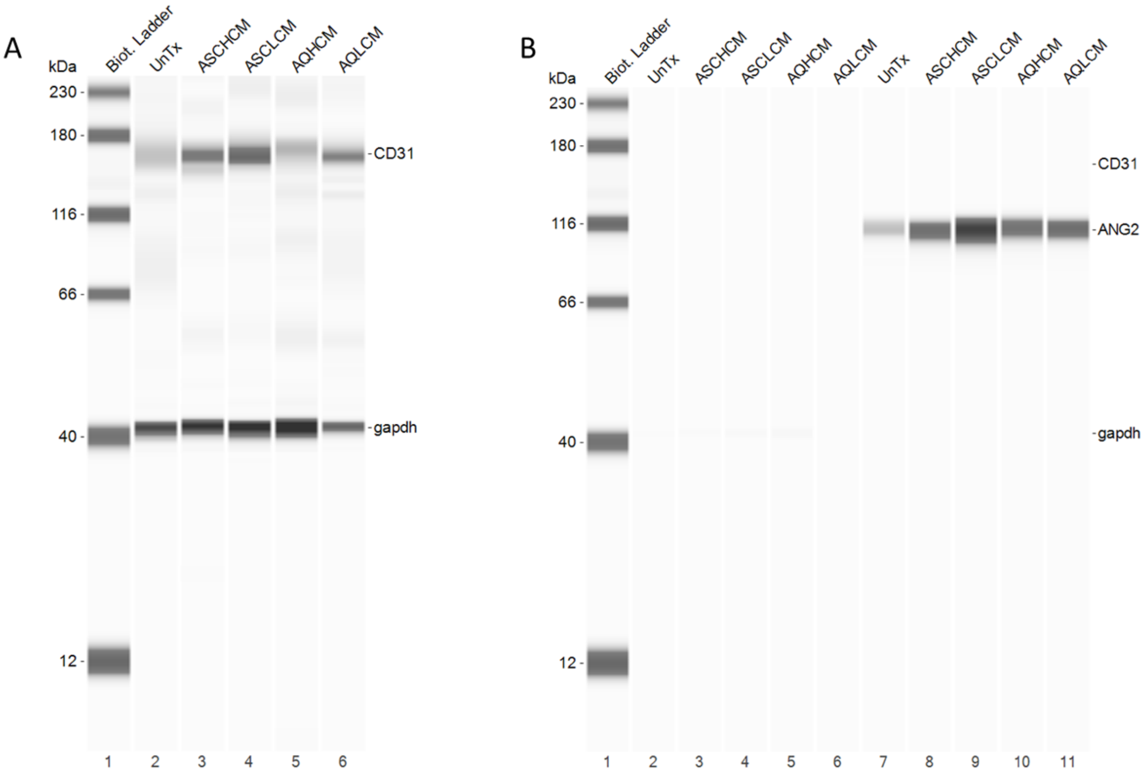

Figure S5. Western Blot showing the expression of CD31, Angiopoietin (ANG2) and GAPDH in HUVECs treated with conditioned media in 3D culture. The samples were run on a 12-230 kDa capillary kit. A-B are different exposures of the same run.

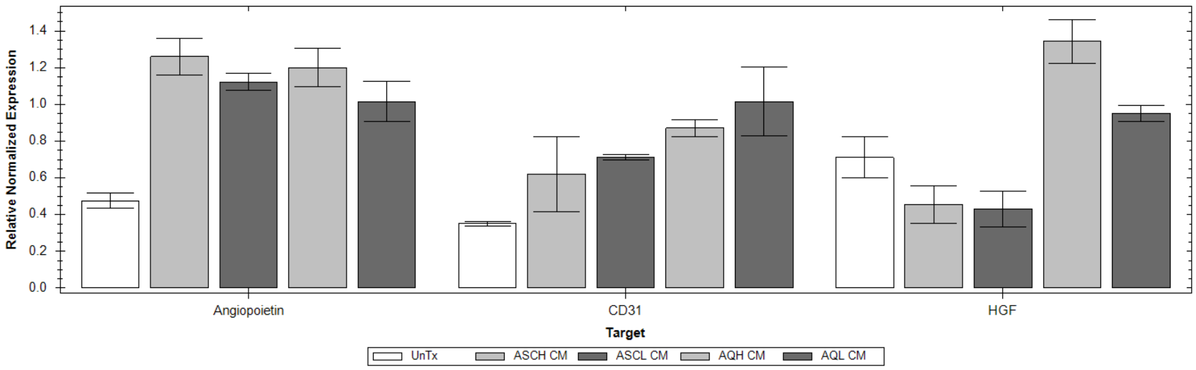

Figure S6. RT-PCR showing increased expression of ANG-2, CD31 and HGF in HUVECs treated cells compared to untreated control cells, 48 h post refeeding with EGM-2 (pooled cells; n = 1).

Table S1. Gene ontology analysis showing the top five biological processes in lipedema ASCs compared to BMI and matched Healthy ASCs (single cells; n=3 per group).

Index	GO Biological Process	p-value	# Genes	Overlap genes
1	Positive regulation of angiogenesis (GO:0045766)	0.0128	13	FOXC2;FLT1;PTGIS;JUP;PIK3CD;VEGFC;PRKCA;HIPK2;BMPER;HYAL1;IL1B;NINJ1;CYP1B1
2	Positive regulation of vasculature development (GO:1904018)	0.0436	11	FLT1;PTGIS;JUP;HYAL1;IL1B;NINJ1;CYP1B1;PIK3CD;VEGFC;PRKCA;HIPK2
3	Regulation of phosphatidylinositol 3-kinase signaling (GO:0014066)	0.0436	11	FLT1;ERBB3;SEMA4D;SRC;ENTPD5;NEDD4;F2R;PIK3CD;F2RL1;PIP4K2C;PIK3AP1
4	Regulation of cell migration (GO:0030334)	0.0436	25	BEX4;SEMA7A;CLIC4;NOTCH1;FLT1;SEMA3C;SEMA4D;SRC;F2R;NOG;CHRD;PIK3CD;PRKCA;PTPRJ;LAMC2;PTPRK;AMOT;BST2;TMEFF2;TMSB4X;IL1B;DPYSL3;CYP1B1;F2RL1

5	Positive regulation of phosphatidylinositol 3-kinase signaling (GO:0014068)	0.0530	9	FLT1;ERBB3;SEMA4D;SRC;NEDD4;F2R;PIK3CD;F2RL1;PIK3AP1

Table S2. RNA-Seq data showing the expression of angiogenic genes in lipedema ASCs compared to Healthy ASCs (single cells; n=3 per group).

Genes	Fold Change	p-value
CXCL3	-1.029	0.02268
CXCL5	-1.588	0.00043
EFNB2	1.476	1.5E-11
FLT1	-1.147	0.00015
ID1	0.819	1.48E-08
IL1B	-0.703	0.01549
TIMP1	-0.933	0.00057
VEGFC	-0.548	0.00003

Materials and Methods:

Dye transfer assay analysis

For flow analysis, we analyzed the data using BD Accuri C6 plus software. The dye transfer assay was calculated by dividing the mean fluorescent intensity (MFI) of M1 over the percentage of adhered fluorescent cell population after each co-culture. Here are representative images of gated population for dye transfer analysis.

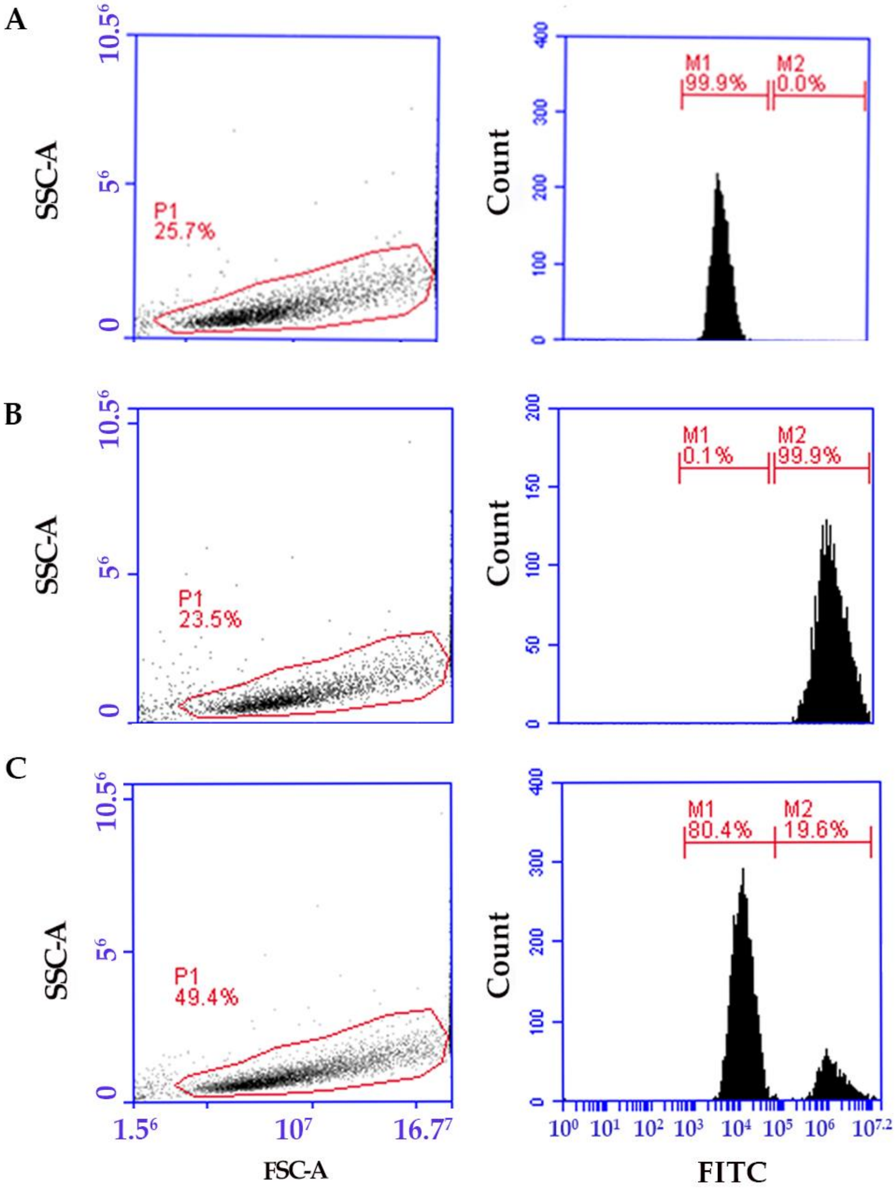

Figure S7. Dye transfer analysis of unlabeled (A), labeled (B) and co-culture (C) cells. P1: represents the population of cells excluding debris. FSC-A: Forward scatter; SSC-A: Side scatter. M1 and M2 are the gates for unlabeled and labelled cells, respectively.
